# Differential game evolution of food quality safety based on market supply and demand

**DOI:** 10.1002/fsn3.2128

**Published:** 2021-03-25

**Authors:** Tingqiang Chen, Jun Zhang, Jun Luo

**Affiliations:** ^1^ School of Economics and Management Nanjing Tech University Nanjing China; ^2^ School of Health Economics and Management Nanjing University of Chinese Medicine Nanjing China

**Keywords:** decision‐making situations, differential games, food quality, market supply and demand

## Abstract

Frequent outbreaks of food quality and safety problems have seriously damaged the interests of consumers and reduced their confidence in China's food safety. In this study based on market supply and demand, we design a differential game model between food supplier and food retailer by considering different decision‐making situations. We also analyze the optimal revenue of the food supplier and food retailer on food quality efforts, the overall return of the supply chain, the level of food quality and safety, and their evolutionary characteristics. Results of the analysis indicate the following. (a) From the situation of decentralized decision‐making to the situation of decision‐making under the incentive strategy, a Pareto improvement occurs in the food quality and safety strategy of food supplier, food retailer, and even the entire food supply chain. (b) The optimal revenues of the supplier and retailer, overall supply chain revenue, and efforts of the supplier and retailer are all affected by changes in market supply and demand, resulting in drastic fluctuations. On the whole, food quality tends to improve over time and will fluctuate slightly due to changes in market supply and demand. (c) If the market supply is stable when supply exceeds demand and the market demand turns from a downward trend to an upward trend, then food quality safety risk will be higher than in other periods.

## Graphical abstract

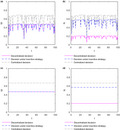

## 1 INTRODUCTION

With the continuous development of our social economy, public demand for the quality and safety of food safety has also improved, which poses challenges for food quality and safety management. In this context, food quality safety incidents frequently occur, such as the melamine milk incident, the gutter oil incident, and the Clenbuterol incident. The significant impact of these food safety incidents not only harms the health of consumers but also reduces their confidence in China's food safety (Liu et al., 2018; Lu & Wu, 2014; Pei et al., 2011).

To cope with the increasingly serious food safety problems, scholars have conducted extensive studies on food safety risks, and their main research interests include the analysis and evaluation of the food safety regulatory system and food safety policies (Antle, 1999; Jia & Jukes, 2013; KöNig, 2007; Starbird, 2005; Worosz et al., 2008). Studies have also been conducted on the role of third‐party institutions in the existing safety supervision system (Albersmeier et al., 2009; Tanner, 2000; Turku et al., 2018; Zhang et al., 2015), consumer attitude toward food safety (Jevšnik et al., 2008; Mascarello et al., 2015; Tiozzo et al., 2017; Wilcock et al., 2004; Zhang et al., 2018), and the assessment of food safety risk status (Li et al., 2020; Ross & Sumner, 2002; Stadlmüller et al., 2017; Tutu & Anfu, 2019; Yang et al., 2019). In his study on the food safety regulatory system and food safety policies, Antle (1999) provided a framework for measuring the benefits and costs of food safety regulations and proposed suggestions for future quantitative studies on the benefits and costs of food safety regulations. In their study on the role of third‐party institutions in the existing safety supervision system, Turku et al. (2018) investigated the consistency between the official inspection results of food enterprises and the audit results of third‐party institutions. However, although most people believe that the results of the two aforementioned studies overlap, significant differences are still apparent. In their research on consumer attitudes toward food safety, Zhang et al. (2018) discussed the factors influencing the willingness of consumers to buy safe vegetables and found that consumers believe the main advantage of safe vegetables is the strict production and processing environment of food safety. In their study on assessing the risk status of food safety, Yang et al. (2019) used the Bayesian probability model and meta‐analysis technology to evaluate the overall quality and safety status of Chinese food. Their results showed that 98.26% of the food in the market met the relevant food quality and safety standards. Dairy products and food additives have the lowest risk of food quality and safety. Beverages, alcohols, processed fruit and vegetable products, and banquet food have a higher risk of food quality and safety.

However, only through understanding the influencing factors of food quality and the occurrence mechanism of food safety incidents can we better seek the countermeasures of food safety supervision (Millstone, 2007). Consequently, such factors and occurrence mechanism have gradually attracted the attention of scholars. Scholars have found that the capital investment ability of enterprises (Chen et al., 2018), transparency in the supply chain of food enterprises (Beulens et al., 2005), and enterprise scale (Dora et al., 2013), among other factors, all have an impact on food safety management. For example, regarding the relationship between enterprise scale and the state of food quality management, Dora et al. (2013) believes that medium‐sized enterprises are more proficient than small enterprises in the application of a food quality management system. Jiang and Batt (2016) also believe that most small enterprises lack financial resources and infrastructure, making it difficult for them to pursue food safety management. Meanwhile, Rouvière (2016) put forward different viewpoints because he found that when cross‐contamination is impossible, small companies tend to take stronger preventive measures than do large companies, and when cross‐contamination is possible, both large and small companies spend less on prevention than do medium‐sized companies. Thus, the relationship between food quality and the size of food enterprises is not linear.

With the deepening of research on food quality and safety, scholars have gradually found that the changes in market supply and market demand will have an impact on food quality and safety. Lin (2017) believes that the shortage of productivity is the biggest threat to food safety, partly reflecting that the shortage of food supply will lead to the decline of food quality. Wang and Chen (2016) find that with the increase of the imbalance between supply and demand, the risk diffusion rate of unsafe food will increase rapidly. However, existing research rarely involves an analysis on the influence of market supply and market demand changes on the evolution of food quality, a knowledge gap that is not conducive to the supervision of food enterprises and the guarantee of food safety. Therefore, this work will analyze the efforts in food quality of the food supplier and retailer, the optimal revenue of the retailer and supplier and the overall revenue of supply chain under market supply and demand changes in different decision‐making situations. The ultimate goal is to understand the evolution of food quality in terms of the change in the market supply and demand situation and how the decision‐making situations of food enterprises affect food quality evolution.

The rest of this paper is organized as follows. The second section defines the income and cost functions of food enterprises and constructs the food quality and safety model. The third section characterizes the supplier's and retailer's efforts and revenue, total revenue of the supply chain, and food quality and safety level as a function of time under different decision‐making situations. The fourth section compares and analyzes the above functions in different decision‐making situations. The fifth section analyzes the evolution of the supplier and retailer under different decision situations and parameter changes through a computation experiment and a simulation of the model. The sixth section concludes this paper.

## 2 PROBLEM DESCRIPTION

In this study, the main players of the game are the food supplier and the retailer, who are rational participants in the food market and take profit maximization as the decision‐making goal. We assume that the efforts of the supplier and retailer in food quality and safety are M(t) and N(t), respectively, and their cost functions in food quality and safety are $C_{M}\left(t\right)$ and $C_{N}\left(t\right)$, respectively. Generally, the cost of efforts gradually increases with the increase of food quality. Suppose the cost functions of the supplier and retailer in food quality and safety are $C_{M}\left(t\right)=\frac{1}{2}k_{1}M^{2}\left(t\right)$ and $C_{N}\left(t\right)=\frac{1}{2}k_{2}N^{2}\left(t\right)$, respectively, where $k_{1}$ and $k_{2}$ are the cost coefficients for food quality and safety efforts by the supplier and retailer, respectively.

Given that food quality and safety require the accumulation of efforts over a long period of time, food quality can be considered as the accumulated result of the supplier's and retailer's effort in food quality and safety. The accumulated efforts in food quality per unit product of the supplier and retailer follow the following motion equation:

(1) $$R^{\prime }\left(t\right)=k_{3}M\left(t\right)+k_{4}N\left(t\right)‐k_{5}R\left(t\right),$$

where R(t) represents food quality at time t, with the high value of R(t) representing high food quality at time t; and $R_{0}$ represents food quality when t=0. $k_{3}$ is the influence coefficient of the supplier's efforts in food quality and safety on food quality per unit product. $k_{4}$ is the influence coefficient of the retailer's efforts in food quality and safety on food quality per product. $k_{5}$ is the relative attenuation rate of the quality function when both supplier and retailer do not make efforts in food quality safety, $0<k_{5}<1$.

The market price of food is influenced by several factors. On the one hand, it is influenced by the quality of food, with consumers willing to pay higher prices for high‐quality food (Batt & Parining, 2000; Wang et al., 2020). On the other hand, it is influenced by market supply and demand. On the basis of the above analysis, the market price of food is assumed to be correlated linearly with food quality, and the food price function is constructed as follows:

(2) P(t)=R(t)p(t),

where P(t) is the market price of food at time t, and p(t) represents the basic price at time t when the food quality is 1. The basic price p(t) is only affected by the market supply and demand at time t.

The market price of food is affected by the total market supply and demand, and its function relation, which is referred to in Caginalp and Caginalp (2019), is used to describe the price change function of frequently traded products:

(3) $$\frac{dp(t)}{\mathrm{dt}}=k_{6}\left(\frac{Q(t)‐S(t)}{S(t)}\right)p\left(t\right).$$

By solving Equation (3) through solving differential equations, we can obtain Equation (4) as follows:

(4) $$p\left(t\right)=p_{0}e^{k_{6}\int \left(\frac{Q(t)‐S(t)}{S(t)}\right)dt},$$

where $p_{0}$ represents the basic price at time t=0. In this study, the market supply and market demand curves are determined by the market rather than by the food supplier. The food supplier and retailer need to make decisions about their degree of food quality effort according to the market environment, including supply and demand conditions. $k_{6}$ is a constant coefficient that measures the influence of food price on market supply and demand. The higher the $k_{6}$ value is, the greater the influence of food price on market supply and market demand will be.

According to Equations (2) and (4), the food price function is constructed as follows:

(5) $$P\left(t\right)=R\left(t\right)p_{0}e^{k_{6}\int \left(\frac{Q(t)‐S(t)}{S(t)}\right)dt}.$$

The sales quantity of food can be considered as the smaller value of the enterprise's food production and market demand. Thus, the income function of food is

(6) $$I\left(t\right)=\min\left\{Q(t),S(t)\right\}R\left(t\right)p_{0}e^{k_{6}\int \left(\frac{Q(t)‐S(t)}{S(t)}\right)dt}.$$

Moreover, suppose that the cost to the producer to produce the food is

(7) $$C\left(t\right)=k_{7}S\left(t\right),$$

where $k_{7}$ is the production price per unit of food.

The benefits of food are distributed between the supplier and retailer, and the income distribution coefficients of the supplier and retailer are θ and 1‐θ, respectively, and θ∈(0,1). The supplier and retailer have the same discount rates at λ and λ>0, with both sides seeking their own profit maximization strategies within an infinite range.

## 3 DIFFERENTIAL GAME MODEL OF FOOD QUALITY AND SAFETY

### 3.1 Optimal strategy for supply chain members in the case of decentralized decision‐making

In this case, both the supplier and retailer independently determine their own efforts in food quality and safety. The two parties are on equal footing, and the decision is based on maximizing their own interests. The decision problem of the supplier can be expressed as

(8) $$V_{M}\left(t,R\right)=\max_{M(t)}\int_{0}^{\infty }e^{‐\lambda t}\left[\theta I\left(t\right)‐C_{M}\left(t\right)‐C\left(t\right)\right]dt.$$

The decision problem of the retailer can be expressed as

(9) $$V_{N}\left(t,R\right)=\max_{N(t)}\int_{0}^{\infty }e^{‐\lambda t}\left[\left(1‐\theta \right)I\left(t\right)‐C_{N}\left(t\right)\right]dt.$$

Referring to the treatment method of Jørgensen et al. (2003), we assume that the dynamic parameters in the model are constants independent of time. We likewise assume that both the supplier and retailer can realize the maximum benefit functions $V_{M}\left(R\right)$ and $V_{N}\left(R\right)$, and the functions are continuously bounded and differentiable. The optimal strategy combination of the supplier and retailer is the static feedback Nash equilibrium and the Hamilton–Jacobi–Bellman (HJB) equation that meets the requirements of the supplier and retailer are as follows:

(10) $$\lambda V_{M}\left(R\right)=\max_{M}\left\{\theta I\left(t\right)‐C_{M}\left(t\right)‐C\left(t\right)+V_{M}^{\prime }\left(R\right)R^{\prime }\right\}$$

and

(11) $$\lambda V_{N}\left(R\right)=\max_{N}\left\{\left(1‐\theta \right)I\left(t\right)‐C_{N}\left(t\right)+V_{N}^{\prime }\left(R\right)R^{\prime }\right\}.$$

Equations (10) and (11) can be expanded respectively as

(12) $$\lambda V_{M}\left(R\right)=\max_{M}\left\{\theta \min\left\{Q,S\right\}Rp_{0}e^{k_{6}\int \left(\frac{Q‐S}{S}\right)dt}‐k_{7}S‐\frac{1}{2}k_{1}M^{2}+V_{M}^{\prime }\left(R\right)\left(k_{3}M+k_{4}N‐k_{5}R\right)\right\}$$

and

(13) $$\lambda V_{N}\left(R\right)=\max_{N}\left\{\left(1‐\theta \right)\min\left\{Q,S\right\}Rp_{0}e^{k_{6}\int \left(\frac{Q‐S}{S}\right)dt}‐\frac{1}{2}k_{2}N^{2}+V_{N}^{\prime }\left(R\right)\left(k_{3}M+k_{4}N‐k_{5}R\right)\right\}.$$

The first partial derivatives of Equations (12) and (13) with respect to M and N, respectively, are obtained, and the first partial derivative is set as zero. The following equations can then be obtained as

(14) $$M=\frac{k_{3}V_{M}^{\prime }\left(R\right)}{k_{1}}$$

and

(15) $$N=\frac{k_{4}V_{N}^{\prime }\left(R\right)}{k_{2}}.$$

Substitute Equations (14) and (15) into Equations (12) and (13), respectively, to obtain the following equations:

(16) $$\lambda V_{M}\left(R\right)=\theta \min\left\{Q,S\right\}Rp_{0}e^{k_{6}\int \left(\frac{Q‐S}{S}\right)dt}‐k_{7}S‐\frac{k_{3}^{2}{\left(V_{M}^{\prime }\left(R\right)\right)}^{2}}{2k_{1}}+V_{M}^{\prime }\left(R\right)\left(\frac{k_{3}^{2}V_{M}^{\prime }\left(R\right)}{k_{1}}+\frac{k_{4}^{2}V_{N}^{\prime }\left(R\right)}{k_{2}}‐k_{5}R\right)$$

and

(17) $$\lambda V_{N}\left(R\right)=\left(1‐\theta \right)\min\left\{Q,S\right\}Rp_{0}e^{k_{6}\int \left(\frac{Q‐S}{S}\right)dt}‐\frac{k_{4}^{2}{\left(V_{N}^{\prime }\left(R\right)\right)}^{2}}{2k_{2}}+V_{N}^{\prime }\left(R\right)\left(\frac{k_{3}^{2}V_{M}^{\prime }\left(R\right)}{k_{1}}+\frac{k_{4}^{2}V_{N}^{\prime }\left(R\right)}{k_{2}}‐k_{5}R\right).$$

Equation (16) can be simplified to

(18) $$\lambda V_{M}\left(R\right)‐\frac{k_{3}^{2}{\left[V_{M}^{\prime }\left(R\right)\right]}^{2}}{2k_{1}}‐\frac{k_{4}^{2}V_{N}^{\prime }\left(R\right)V_{M}^{\prime }\left(R\right)}{k_{2}}+k_{5}RV_{M}^{\prime }\left(R\right)=\theta \min\left\{Q,S\right\}Rp_{0}e^{k_{6}\int \left(\frac{Q‐S}{S}\right)dt}‐k_{7}S.$$

Equation (17) can be simplified to

(19) $$\lambda V_{N}\left(R\right)‐\frac{k_{4}^{2}{\left[V_{N}^{\prime }\left(R\right)\right]}^{2}}{2k_{2}}‐\frac{k_{3}^{2}V_{M}^{\prime }\left(R\right)V_{N}^{\prime }\left(R\right)}{k_{1}}+k_{5}RV_{N}^{\prime }\left(R\right)=\left(1‐\theta \right)\min\left\{Q,S\right\}Rp_{0}e^{k_{6}\int \left(\frac{Q‐S}{S}\right)dt}.$$

According to the structural characteristics of Equations (18) and (19), the optimal revenue functions $V_{M}\left(R\right)$ and $V_{N}\left(R\right)$ should be linear functions of R, assuming that the functions are

(20) $$V_{M}\left(R\right)=a_{1}R+a_{2}$$

and

(21) $$V_{N}\left(R\right)=b_{1}R+b_{2}.$$

Substitute Equations (20) and (21) into Equations (18) and (19), respectively, to obtain the following equations:

(22) $$a_{1}\lambda R+a_{1}k_{5}R‐\theta \min\left\{Q,S\right\}R\frac{p_{0}}{e^{k_{6}{\left(\int \frac{Q}{S}dt\right|}_{t=0}}}e^{k_{6}\int \left(\frac{Q‐S}{S}\right)dt}+k_{7}S+a_{2}\lambda ‐\frac{a_{1}^{2}k_{3}^{2}}{2k_{1}}‐\frac{a_{1}b_{1}k_{4}^{2}}{k_{2}}=0$$

and

(23) $$b_{1}\lambda R+k_{5}b_{1}R‐\left(1‐\theta \right)\min\left\{Q,S\right\}R\frac{p_{0}}{e^{k_{6}{\left(\int \frac{Q}{S}dt\right|}_{t=0}}}e^{k_{6}\int \left(\frac{Q‐S}{S}\right)dt}+b_{2}\lambda ‐\frac{b_{1}^{2}k_{4}^{2}}{2k_{2}}‐\frac{a_{1}b_{1}k_{3}^{2}}{k_{1}}=0.$$

To ensure that Equations (22) and (23) are valid for all R>0, the coefficient values of the first term and the constant term on both sides of the equation should be equal. Therefore, the values of $a_{1}$, $a_{2}$, $b_{1}$, and $b_{2}$ can be obtained as follows:

(24) $$a_{1}=\frac{\theta \min\left\{Q,S\right\}p_{0}e^{k_{6}\int \left(\frac{Q‐S}{S}\right)dt}}{\lambda +k_{5}},$$

(25) $$b_{1}=\frac{\left(1‐\theta \right)\min\left\{Q,S\right\}p_{0}e^{k_{6}\int \left(\frac{Q‐S}{S}\right)dt}}{\lambda +k_{5}},$$

(26) $$a_{2}=\frac{k_{3}^{2}}{2k_{1}\lambda }{\left\{\frac{\theta \min\left\{Q,S\right\}p_{0}e^{k_{6}\int \left(\frac{Q‐S}{S}\right)dt}}{\lambda +k_{5}}\right\}}^{2}+\frac{k_{4}^{2}}{k_{2}\lambda }\left\{\frac{\theta \min\left\{Q,S\right\}p_{0}e^{k_{6}\int \left(\frac{Q‐S}{S}\right)dt}}{\lambda +k_{5}}\right\}\left\{\frac{\left(1‐\theta \right)\min\left\{Q,S\right\}p_{0}e^{k_{6}\int \left(\frac{Q‐S}{S}\right)dt}}{\lambda +k_{5}}\right\}‐\frac{k_{7}S}{\lambda },$$

and

(27) $$b_{2}=\frac{k_{4}^{2}}{2k_{2}\lambda }{\left\{\frac{\left(1‐\theta \right)\min\left\{Q,S\right\}p_{0}e^{k_{6}\int \left(\frac{Q‐S}{S}\right)dt}}{\lambda +k_{5}}\right\}}^{2}+\frac{k_{3}^{2}}{k_{1}\lambda }\left\{\frac{\theta \min\left\{Q,S\right\}p_{0}e^{k_{6}\int \left(\frac{Q‐S}{S}\right)dt}}{\lambda +k_{5}}\right\}\left\{\frac{\left(1‐\theta \right)\min\left\{Q,S\right\}p_{0}e^{k_{6}\int \left(\frac{Q‐S}{S}\right)dt}}{\lambda +k_{5}}\right\}.$$

When Equations (24)–(27) are substituted into Equations (20) and (21), the optimal revenues of the supplier and retailer and the revenue of the supply chain as a whole in the case of decentralized decision‐making, which are denoted by $V_{M}^{*}\left(R\right)$, $V_{N}^{*}\left(R\right)$, and $V^{*}\left(R\right)$, respectively, can be obtained as follows:

(28) $$\begin{array}{c} V_{M}^{*}\left(R\right)=\frac{\theta \min\left\{Q,S\right\}p_{0}e^{k_{6}\int \left(\frac{Q‐S}{S}\right)dt}}{\lambda +k_{5}}R^{*}+\frac{k_{4}^{2}}{k_{2}\lambda }\left\{\frac{\theta \min\left\{Q,S\right\}p_{0}e^{k_{6}\int \left(\frac{Q‐S}{S}\right)dt}}{\lambda +k_{5}}\right\}\left\{\frac{\left(1‐\theta \right)\min\left\{Q,S\right\}p_{0}e^{k_{6}\int \left(\frac{Q‐S}{S}\right)dt}}{\lambda +k_{5}}\right\}\\ +\frac{k_{3}^{2}}{2k_{1}\lambda }{\left\{\frac{\theta \min\left\{Q,S\right\}p_{0}e^{k_{6}\int \left(\frac{Q‐S}{S}\right)dt}}{\lambda +k_{5}}\right\}}^{2}‐\frac{k_{7}S}{\lambda }, \end{array}$$

(29) $$\begin{array}{c} V_{N}^{*}\left(R\right)=\frac{\left(1‐\theta \right)\min\left\{Q,S\right\}p_{0}e^{k_{6}\int \left(\frac{Q‐S}{S}\right)dt}}{\lambda +k_{5}}R^{*}+\frac{k_{3}^{2}}{k_{1}\lambda }\left\{\frac{\theta \min\left\{Q,S\right\}p_{0}e^{k_{6}\int \left(\frac{Q‐S}{S}\right)dt}}{\lambda +k_{5}}\right\}\left\{\frac{\left(1‐\theta \right)\min\left\{Q,S\right\}p_{0}e^{k_{6}\int \left(\frac{Q‐S}{S}\right)dt}}{\lambda +k_{5}}\right\}\\ +\frac{k_{4}^{2}}{2k_{2}\lambda }{\left\{\frac{\left(1‐\theta \right)\min\left\{Q,S\right\}p_{0}e^{k_{6}\int \left(\frac{Q‐S}{S}\right)dt}}{\lambda +k_{5}}\right\}}^{2}, \end{array}$$

and

(30) $$\begin{array}{c} V^{*}\left(R\right)=\frac{\min\left\{Q,S\right\}p_{0}e^{k_{6}\int \left(\frac{Q‐S}{S}\right)dt}}{\lambda +k_{5}}R^{*}+\frac{k_{3}^{2}}{2k_{1}\lambda }{\left\{\frac{\theta \min\left\{Q,S\right\}p_{0}e^{k_{6}\int \left(\frac{Q‐S}{S}\right)dt}}{\lambda +k_{5}}\right\}}^{2}+\frac{k_{4}^{2}}{2k_{2}\lambda }{\left\{\frac{\left(1‐\theta \right)\min\left\{Q,S\right\}p_{0}e^{k_{6}\int \left(\frac{Q‐S}{S}\right)dt}}{\lambda +k_{5}}\right\}}^{2}\\ +\left(\frac{k_{4}^{2}}{k_{2}\lambda }+\frac{k_{3}^{2}}{k_{1}\lambda }\right)\left\{\frac{\theta \min\left\{Q,S\right\}p_{0}e^{k_{6}\int \left(\frac{Q‐S}{S}\right)dt}}{\lambda +k_{5}}\right\}\left\{\frac{\left(1‐\theta \right)\min\left\{Q,S\right\}p_{0}e^{k_{6}\int \left(\frac{Q‐S}{S}\right)dt}}{\lambda +k_{5}}\right\}‐\frac{k_{7}S}{\lambda }. \end{array}$$

When Equations (28) and (29) are substituted into Equations (14) and (15), the efforts of the supplier and retailer on food quality and safety in the case of decentralized decision‐making, which are denoted by $M^{*}$ and $N^{*}$, respectively, can be obtained as follows:

(31) $$M^{*}=\frac{\theta k_{3}\min\left\{Q,S\right\}p_{0}e^{k_{6}\int \left(\frac{Q‐S}{S}\right)dt}}{\lambda k_{1}+k_{1}k_{5}}$$

and

(32) $$N^{*}=\frac{\left(1‐\theta \right)k_{4}\min\left\{Q,S\right\}p_{0}e^{k_{6}\int \left(\frac{Q‐S}{S}\right)dt}}{\lambda k_{2}+k_{2}k_{5}}.$$

When Equations (31) and (32) are substituted into Equation (1), the evolution trajectory of the food quality change process of unit product in the case of decentralized decision‐making, which is denoted by $R^{*}\left(t\right)$, can be obtained as

(33) $$R^{*}\left(t\right)=R_{0}e^{‐k_{5}t}+e^{‐k_{5}t}\left[\frac{\theta k_{3}^{2}}{\lambda k_{1}+k_{1}k_{5}}+\frac{\left(1‐\theta \right)k_{4}^{2}}{k_{2}\left(\lambda +k_{5}\right)}\right]\int \min\left\{Q,S\right\}p_{0}e^{k_{6}\int \left(\frac{Q‐S}{S}\right)dt+k_{5}t}dt,$$

where $R_{0}$ is the food quality at time t=0.

### 3.2 Optimal strategy for supply chain members in the case of decision‐making under the supplier incentive strategy

In this case, the supplier provides incentive policies for the retailer's food safety input, subsidizes the retailer's food quality input cost, and bears a certain proportion of the retailer's food quality and safety effort cost. Suppose the ratio of the supplier to bear the effort cost in food quality and safety is η, and 1>η>0. The decision problem of the supplier can be expressed as

(34) $$V_{M}\left(t,R\right)=\max_{M\left(t\right)}\int_{0}^{\infty }e^{‐\lambda t}\left[\theta I\left(t\right)‐C_{M}\left(t\right)‐\eta C_{N}\left(t\right)‐C\left(t\right)\right]dt.$$

The decision problem of the retailer can be expressed as

(35) $$V_{N}\left(t,R\right)=\max_{N\left(t\right)}\int_{0}^{\infty }e^{‐\lambda t}\left[\left(1‐\theta \right)I\left(t\right)‐\left(1‐\eta \right)C_{N}\left(t\right)\right]dt.$$

The supplier is the leader of the game and the retailer is the follower of the game, and both play a Stackelberg game. First, the supplier determines the effort in food quality and safety and the proportion of the supplier's effort cost in food quality and safety for the retailer, and then the retailer determines the effort in food quality and safety. Both parties take the maximization of their own interests as the decision‐making objective. Therefore, the backward induction method is used to solve the unilateral optimal control problem of the retailer, and the HJB equation is

(36) $$\lambda V_{N}\left(R\right)=\max_{N}\left\{\left(1‐\theta \right)I\left(t\right)‐\left(1‐\eta \right)C_{N}\left(t\right)+V_{N}^{\prime }\left(R\right)R^{\prime }\right\}.$$

Equation (36) can be expanded as

(37) $$\lambda V_{N}\left(R\right)=\max_{N}\left\{\left(1‐\theta \right)\min\left\{Q,S\right\}Rp_{0}e^{k_{6}\int \left(\frac{Q‐S}{S}\right)dt}‐\left(1‐\eta \right)\frac{1}{2}k_{2}N^{2}+V_{N}^{\prime }\left(R\right)\left(k_{3}M+k_{4}N‐k_{5}R\right)\right\}.$$

The first partial derivative of Equation (37) with respect to N is set as zero and then the following equation can be obtained as

(38) $$N=\frac{k_{4}V_{N}^{\prime }\left(R\right)}{\left(1‐\eta \right)k_{2}}.$$

The supplier predicts that the retailer will take Equation (38) as its strategy for food quality and safety, so the supplier will determine its own optimal strategy based on Equation (38). The HJB equation of the supplier will be

(39) $$\lambda V_{M}\left(R\right)=\max_{M}\left\{\theta \min\left\{Q,S\right\}Rp_{0}e^{k_{6}\int \left(\frac{Q‐S}{S}\right)dt}‐k_{7}S‐\frac{1}{2}k_{1}M^{2}‐\eta \frac{1}{2}k_{2}N^{2}+V_{M}^{\prime }\left(R\right)\left(k_{3}M+k_{4}N‐k_{5}R\right)\right\}.$$

The new equation can be obtained by substituting Equation (38) into Equation (39) and then the first partial derivative with respect to M and η of the new equation can be obtained. Next, the first partial derivatives are set as zero, and the following equations can be obtained as

(40) $$\eta =\frac{2V_{M}^{\prime }\left(R\right)‐V_{N}^{\prime }\left(R\right)}{V_{N}^{\prime }\left(R\right)+2V_{M}^{\prime }\left(R\right)}$$

and

(41) $$M=\frac{k_{3}V_{M}^{\prime }\left(R\right)}{k_{1}}.$$

When Equations (38) and (41) are substituted into Equations (37) and (39), the following equations can be obtained as

(42) $$\lambda V_{N}\left(R\right)‐\frac{k_{4}^{2}{\left(V_{N}^{\prime }\left(R\right)\right)}^{2}+2k_{4}^{2}V_{M}^{\prime }\left(R\right)V_{N}^{\prime }\left(R\right)}{4k_{2}}‐\frac{k_{3}^{2}V_{M}^{\prime }\left(R\right)V_{N}^{\prime }\left(R\right)}{k_{1}}+k_{5}RV_{N}^{\prime }\left(R\right)=(1‐\theta )\min\left\{Q,S\right\}Rp_{0}e^{k_{6}\int \left(\frac{Q‐S}{S}\right)dt}$$

and

(43) $$\lambda V_{M}\left(R\right)‐\frac{k_{3}^{2}{\left(V_{M}^{\prime }\left(R\right)\right)}^{2}}{2k_{1}}‐\frac{k_{4}^{2}{\left(V_{M}^{\prime }\left(R\right)\right)}^{2}}{2k_{2}}‐\frac{k_{4}^{2}{\left(V_{N}^{\prime }\left(R\right)\right)}^{2}}{8k_{2}}‐\frac{k_{4}^{2}V_{M}^{\prime }\left(R\right)V_{N}^{\prime }\left(R\right)}{2k_{2}}+k_{5}RV_{M}^{\prime }\left(R\right)=\theta \min\left\{Q,S\right\}Rp_{0}e^{k_{6}\int \left(\frac{Q‐S}{S}\right)dt}‐k_{7}S.$$

According to the structural characteristics of Equations (42) and (43), it is speculated that the optimal revenue functions $V_{M}\left(R\right)$ and $V_{N}\left(R\right)$ should be linear functions of R, assuming that the functions are

(44) $$V_{M}\left(R\right)\left(R\right)=a_{1}R+a_{2}$$

and

(45) $$V_{N}\left(R\right)=b_{1}R+b_{2}.$$

When Equations (44) and (45) are substituted into Equations (42) and (43), the following equations can be obtained as

(46) $$b_{1}\lambda R‐\left(1‐\theta \right)\min\left\{Q,S\right\}Rp_{0}e^{k_{6}\int \left(\frac{Q‐S}{S}\right)dt}+b_{1}k_{5}R‐\left(\frac{k_{4}^{2}b_{1}^{2}+2a_{1}b_{1}k_{4}^{2}}{4k_{2}}+\frac{k_{3}^{2}a_{1}b_{1}}{k_{1}}‐b_{2}\lambda \right)=0$$

and

(47) $$a_{1}\lambda R‐\theta \min\left\{Q,S\right\}Rp_{0}e^{k_{6}\int \left(\frac{Q‐S}{S}\right)dt}+k_{7}S+a_{1}k_{5}R‐\left(\frac{k_{3}^{2}a_{1}^{2}}{2k_{1}}+\frac{k_{4}^{2}a_{1}^{2}}{2k_{2}}+\frac{k_{4}^{2}b_{1}^{2}}{8k_{2}}+\frac{k_{4}^{2}a_{1}b_{1}}{2k_{2}}‐a_{2}\lambda \right)=0.$$

To ensure that Equations (46) and (47) are valid for all R>0, the coefficient values of the first term and the constant term on both sides of the equation should be equal. Hence, the values of $a_{1}$, $a_{2}$, $b_{1}$, and $b_{2}$ can be obtained as follows:

(48) $$a_{1}=\frac{\theta k_{6}\min\left\{Q,S\right\}p_{0}e^{k_{6}\int \left(\frac{Q‐S}{S}\right)dt}}{\lambda +k_{5}},$$

(49) $$b_{1}=\frac{\left(1‐\theta \right)\min\left\{Q,S\right\}p_{0}e^{k_{6}\int \left(\frac{Q‐S}{S}\right)dt}}{\lambda +k_{5}},$$

(50) $$\begin{array}{c} a_{2}=\left(\frac{k_{3}^{2}}{2\lambda k_{1}}+\frac{k_{4}^{2}}{2\lambda k_{2}}\right){\left\{\frac{\theta \min\left\{Q,S\right\}p_{0}e^{k_{6}\int \left(\frac{Q‐S}{S}\right)dt}}{\lambda +k_{5}}\right\}}^{2}+\frac{k_{4}^{2}}{8\lambda k_{2}}{\left\{\frac{\left(1‐\theta \right)\min\left\{Q,S\right\}p_{0}e^{k_{6}\int \left(\frac{Q‐S}{S}\right)dt}}{\lambda +k_{5}}\right\}}^{2}\\ +\frac{k_{4}^{2}}{2\lambda k_{2}}\left\{\frac{\theta \min\left\{Q,S\right\}p_{0}e^{k_{6}\int \left(\frac{Q‐S}{S}\right)dt}}{\lambda +k_{5}}\right\}\left\{\frac{\left(1‐\theta \right)\min\left\{Q,S\right\}p_{0}e^{k_{6}\int \left(\frac{Q‐S}{S}\right)dt}}{\lambda +k_{5}}\right\}‐\frac{k_{7}S}{\lambda }, \end{array}$$

and

(51) $$b_{2}=\frac{k_{4}^{2}}{4\lambda k_{2}}{\left\{\frac{\left(1‐\theta \right)\min\left\{Q,S\right\}p_{0}e^{k_{6}\int \left(\frac{Q‐S}{S}\right)dt}}{\lambda +k_{5}}\right\}}^{2}+\left(\frac{2k_{4}^{2}}{4\lambda k_{2}}+\frac{k_{3}^{2}}{k_{1}\lambda }\right)\left\{\frac{\theta \min\left\{Q,S\right\}p_{0}e^{k_{6}\int \left(\frac{Q‐S}{S}\right)dt}}{\lambda +k_{5}}\right\}\left\{\frac{\left(1‐\theta \right)\min\left\{Q,S\right\}p_{0}e^{k_{6}\int \left(\frac{Q‐S}{S}\right)dt}}{\lambda +k_{5}}\right\}.$$

When Equations (48)–(51) are substituted into Equations (40)–(45), the optimal revenues of the supplier and retailer and the revenue of the supply chain as a whole in the case of decision‐making under the incentive strategy with the optimal cost assumption coefficient, which are denoted by $V_{M}^{**}\left(R\right)$, $V_{N}^{**}\left(R\right)$, and $V^{**}\left(R\right)$, respectively, can be obtained as follows:

(52) $$\eta =\frac{3\theta ‐1}{1+\theta },$$

(53) $$\begin{array}{c} V_{M}^{**}\left(R\right)=\frac{\theta \min\left\{Q,S\right\}p_{0}e^{k_{6}\int \left(\frac{Q‐S}{S}\right)dt}}{\lambda +k_{5}}R^{**}+\left(\frac{k_{3}^{2}}{2\lambda k_{1}}+\frac{k_{4}^{2}}{2\lambda k_{2}}\right){\left\{\frac{\theta \min\left\{Q,S\right\}p_{0}e^{k_{6}\int \left(\frac{Q‐S}{S}\right)dt}}{\lambda +k_{5}}\right\}}^{2}+\frac{k_{4}^{2}}{8\lambda k_{2}}{\left\{\frac{\left(1‐\theta \right)\min\left\{Q,S\right\}p_{0}e^{k_{6}\int \left(\frac{Q‐S}{S}\right)dt}}{\lambda +k_{5}}\right\}}^{2}\\ +\frac{k_{4}^{2}}{2\lambda k_{2}}\left\{\frac{\theta \min\left\{Q,S\right\}p_{0}e^{k_{6}\int \left(\frac{Q‐S}{S}\right)dt}}{\lambda +k_{5}}\right\}\left\{\frac{\left(1‐\theta \right)\min\left\{Q,S\right\}p_{0}e^{k_{6}\int \left(\frac{Q‐S}{S}\right)dt}}{\lambda +k_{5}}\right\}‐\frac{k_{7}S}{\lambda }, \end{array}$$

(54) $$\begin{array}{c} V_{N}^{**}\left(R\right)=\frac{\left(1‐\theta \right)\min\left\{Q,S\right\}p_{0}e^{k_{6}\int \left(\frac{Q‐S}{S}\right)dt}}{\lambda +k_{5}}R^{**}+\left(\frac{k_{4}^{2}}{2\lambda k_{2}}+\frac{k_{3}^{2}}{k_{1}\lambda }\right)\left\{\frac{\theta \min\left\{Q,S\right\}p_{0}e^{k_{6}\int \left(\frac{Q‐S}{S}\right)dt}}{\lambda +k_{5}}\right\}\left\{\frac{\left(1‐\theta \right)\min\left\{Q,S\right\}p_{0}e^{k_{6}\int \left(\frac{Q‐S}{S}\right)dt}}{\lambda +k_{5}}\right\}\\ +\frac{k_{4}^{2}}{4\lambda k_{2}}{\left\{\frac{\left(1‐\theta \right)\min\left\{Q,S\right\}p_{0}e^{k_{6}\int \left(\frac{Q‐S}{S}\right)dt}}{\lambda +k_{5}}\right\}}^{2}, \end{array}$$

and

(55) $$\begin{array}{c} V^{**}\left(R\right)=\frac{\min\left\{Q,S\right\}p_{0}e^{k_{6}\int \left(\frac{Q‐S}{S}\right)dt}}{\lambda +k_{5}}R^{**}+\left(\frac{k_{3}^{2}}{2\lambda k_{1}}+\frac{k_{4}^{2}}{2\lambda k_{2}}\right){\left\{\frac{\theta \min\left\{Q,S\right\}p_{0}e^{k_{6}\int \left(\frac{Q‐S}{S}\right)dt}}{\lambda +k_{5}}\right\}}^{2}+\frac{3k_{4}^{2}}{8\lambda k_{2}}{\left\{\frac{\left(1‐\theta \right)\min\left\{Q,S\right\}p_{0}e^{k_{6}\int \left(\frac{Q‐S}{S}\right)dt}}{\lambda +k_{5}}\right\}}^{2}\\ +\left(\frac{k_{4}^{2}}{\lambda k_{2}}+\frac{k_{3}^{2}}{k_{1}\lambda }\right)\left\{\frac{\theta \min\left\{Q,S\right\}p_{0}e^{k_{6}\int \left(\frac{Q‐S}{S}\right)dt}}{\lambda +k_{5}}\right\}\left\{\frac{\left(1‐\theta \right)\min\left\{Q,S\right\}p_{0}e^{k_{6}\int \left(\frac{Q‐S}{S}\right)dt}}{\lambda +k_{5}}\right\}‐\frac{k_{7}S}{\lambda }. \end{array}$$

When Equations (53) and (54) are substituted into Equations (38) and (41), the efforts of the supplier and retailer on food quality and safety in the case of decision‐making under the incentive strategy, which are denoted by $M^{**}$ and $N^{**}$, respectively, can be obtained as follows:

(56) $$M^{**}=\frac{\theta k_{3}\min\left\{Q,S\right\}\left[p_{0}‐k_{6}\left(S‐Q\right)\right]}{\lambda k_{1}+k_{1}k_{5}}$$

and

(57) $$N^{**}=\frac{\left(1‐\theta \right)k_{4}\min\left\{Q,S\right\}\left[p_{0}‐k_{6}\left(S‐Q\right)\right]}{\left(1‐\eta \right)k_{2}\left(\lambda +k_{5}\right)}.$$

When Equations (56) and (57) are substituted into Equation (1), the evolution trajectory of food quality change process of unit product in the case of decision‐making under the incentive strategy, which is denoted by $R^{**}\left(t\right)$, can be obtained as

(58) $$R^{**}\left(t\right)=R_{0}e^{‐k_{5}t}+e^{‐k_{5}t}\left[\frac{\theta k_{3}^{2}}{\lambda k_{1}+k_{1}k_{5}}+\frac{\left(1‐\theta \right)k_{4}^{2}}{\left(1‐\eta \right)k_{2}\left(\lambda +k_{5}\right)}\right]\int \min\left\{Q,S\right\}p_{0}e^{k_{6}\int \left(\frac{Q‐S}{S}\right)dt+k_{5}t}dt.$$

### 3.3 Optimal strategy for supply chain members in the case of centralized decision‐making

In this case, the supplier and retailer as a whole determine the efforts of the two sides in food quality and safety strategy, with the overall interests of the food supply chain as the decision‐making objective. The overall decision‐making problem of the food supply chain can be expressed as

(59) $$V\left(t,R\right)=\max_{M(t),P(t)}\int_{0}^{\infty }e^{‐\lambda t}\left[I‐C_{M}\left(t\right)‐C_{N}\left(t\right)‐C\left(t\right)\right]dt.$$

The HJB equation satisfied by the optimal control problem of the food supply chain is

(60) $$\lambda V\left(R\right)=\max_{M,P}\left\{I‐C_{M}\left(t\right)‐C_{N}\left(t\right)‐C\left(t\right)+V_{M}^{\prime }\left(R\right)R^{\prime }\right\}.$$

Equation (60) can be expanded as follows:

(61) $$\lambda V\left(R\right)=\min\left\{Q,S\right\}Rp_{0}e^{k_{6}\int \left(\frac{Q‐S}{S}\right)dt}‐k_{7}S‐\frac{1}{2}k_{1}M^{2}‐\frac{1}{2}k_{2}N^{2}+V^{\prime }\left(R\right)\left(k_{3}M+k_{4}N‐k_{5}R\right).$$

The first partial derivatives of Equation (61) with respect to M and N are obtained, and the first partial derivatives are set as zero. The following equations can then be obtained as

(62) $$M=\frac{k_{3}V^{\prime }\left(R\right)}{k_{1}}$$

and

(63) $$N=\frac{k_{4}V^{\prime }\left(R\right)}{k_{2}}.$$

When Equations (62) and (63) are substituted into Equation (61), the following equations can be obtained:

(64) $$\lambda V\left(R\right)‐\frac{k_{3}^{2}{\left[V^{\prime }\left(R\right)\right]}^{2}}{2k_{1}}‐\frac{k_{4}^{2}{\left[V^{\prime }\left(R\right)\right]}^{2}}{2k_{2}}+k_{5}RV^{\prime }\left(R\right)=\min\left\{Q,S\right\}Rp_{0}e^{k_{6}\int \left(\frac{Q‐S}{S}\right)dt}‐k_{7}S.$$

According to the structural characteristics of Equation (64), it is speculated that the optimal revenue functions V(R) should be the linear functions of R, assuming that the functions are

(65) V(R)=aR+b.

When Equation (65) is substituted into Equation (64), the following equation can be obtained as

(66) $$aR\lambda +ak_{5}R‐\min\left\{Q,S\right\}Rp_{0}e^{k_{6}\int \left(\frac{Q‐S}{S}\right)dt}+k_{7}S+b\lambda ‐\frac{k_{3}^{2}a^{2}}{2k_{1}}‐\frac{k_{4}^{2}a^{2}}{2k_{2}}=0.$$

To ensure that Equation (66) is valid for all R>0, the coefficient values of the first term and the constant term on both sides of the equation should be equal, so the values of a and b can be obtained as follows:

(67) $$a=\frac{\min\left\{Q,S\right\}p_{0}e^{k_{6}\int \left(\frac{Q‐S}{S}\right)dt}}{\lambda +k_{5}}$$

and

(68) $$b=\left(\frac{k_{3}^{2}}{2\lambda k_{1}}+\frac{k_{4}^{2}}{2\lambda k_{2}}\right){\left\{\frac{\min\left\{Q,S\right\}p_{0}e^{k_{6}\int \left(\frac{Q‐S}{S}\right)dt}‐k_{7}S}{\lambda +k_{5}}\right\}}^{2}‐\frac{k_{7}S}{\lambda }.$$

When Equations (67) and (68) are substituted into Equation (65), the optimal revenues of the supplier and retailer and the revenue of the supply chain as a whole in the case of centralized decision‐making with the optimal cost assumption coefficient, which are denoted by $V_{M}^{***}\left(R\right)$, $V_{N}^{***}\left(R\right)$, and $V^{***}\left(R\right)$, respectively, can be obtained as follows:

(69) $$V_{M}^{***}\left(R\right)=\frac{\theta \min\left\{Q,S\right\}p_{0}e^{k_{6}\int \left(\frac{Q‐S}{S}\right)dt}}{\lambda +k_{5}}R^{***}+\frac{k_{3}^{2}}{2\lambda k_{1}}{\left\{\frac{\min\left\{Q,S\right\}p_{0}e^{k_{6}\int \left(\frac{Q‐S}{S}\right)dt}}{\lambda +k_{5}}\right\}}^{2}‐\frac{k_{7}S}{\lambda }.$$

(70) $$V_{N}^{***}\left(R\right)=\frac{\left(1‐\theta \right)\min\left\{Q,S\right\}p_{0}e^{k_{6}\int \left(\frac{Q‐S}{S}\right)dt}}{\lambda +k_{5}}R^{***}+\frac{k_{4}^{2}}{2\lambda k_{2}}{\left\{\frac{\min\left\{Q,S\right\}p_{0}e^{k_{6}\int \left(\frac{Q‐S}{S}\right)dt}}{\lambda +k_{5}}\right\}}^{2},$$

and

(71) $$V^{***}\left(R\right)=\frac{\min\left\{Q,S\right\}p_{0}e^{k_{6}\int \left(\frac{Q‐S}{S}\right)dt}}{\lambda +k_{5}}R^{***}+\left(\frac{k_{3}^{2}}{2\lambda k_{1}}+\frac{k_{4}^{2}}{2\lambda k_{2}}\right){\left\{\frac{\min\left\{Q,S\right\}p_{0}e^{k_{6}\int \left(\frac{Q‐S}{S}\right)dt}}{\lambda +k_{5}}\right\}}^{2}‐\frac{k_{7}S}{\lambda }.$$

When Equation (71) is substituted into Equations (62) and (63), the efforts adopted by the supplier and retailer on food quality and safety in the case of centralized decision‐making, which are denoted by $M^{***}$ and $N^{***}$, respectively, can be obtained as follows:

(72) $$M^{***}=\frac{k_{3}\min\left\{Q,S\right\}p_{0}e^{k_{6}\int \left(\frac{Q‐S}{S}\right)dt}}{\lambda k_{1}+k_{1}k_{5}}$$

and

(73) $$N^{***}=\frac{k_{4}\min\left\{Q,S\right\}p_{0}e^{k_{6}\int \left(\frac{Q‐S}{S}\right)dt}}{\lambda k_{2}+k_{2}k_{5}}.$$

When Equations (72) and (73) are substituted into Equation (1), the evolution trajectory of food quality change process of unit product in the case of centralized decision‐making, which is denoted by $R^{***}\left(t\right)$, can be obtained as

(74) $$R^{***}\left(t\right)=R_{0}e^{‐k_{5}t}+e^{‐k_{5}t}\left(\frac{k_{3}^{2}}{\lambda k_{1}+k_{1}k_{5}}+\frac{k_{4}^{2}}{\lambda k_{2}+k_{2}k_{5}}\right)\int \min\left\{Q,S\right\}p_{0}e^{k_{6}\int \left(\frac{Q‐S}{S}\right)dt+k_{5}t}dt.$$

## 4 ANALYSIS OF FOOD QUALITY AND SAFETY EQUILIBRIUM RESULTS

### 4.1 Comparison of food quality and safety efforts of the supplier and retailer in different situations

According to Equations (31), (56), and (72), the optimal effort of the supplier in different situations can be compared as follows:

$$M^{**}‐M^{*}=0.$$

and

$$M^{***}‐M^{**}=(1‐\theta )\frac{k_{3}\min\left\{Q,S\right\}p_{0}e^{k_{6}\int \left(\frac{Q‐S}{S}\right)dt}}{\lambda k_{1}+k_{1}k_{5}}=(1‐\theta )M^{***}>0.$$

The supplier's effort in the case of centralized decision‐making is shown to be higher than that in the case of decentralized decision‐making or decision‐making under the incentive strategy. The supplier's effort in the case of decentralized decision‐making is the same as that in the case of decision‐making under the incentive strategy.

According to Equations (32), (57), and (73), the retailer's optimal effort in different situations can be compared as follows:

$$N^{**}‐N^{*}=\left(\frac{\eta }{1‐\eta }\right)\frac{\left(1‐\theta \right)k_{4}\min\left\{Q,S\right\}p_{0}e^{k_{6}\int \left(\frac{Q‐S}{S}\right)dt}}{k_{2}\left(\lambda +k_{5}\right)}=N^{*}\cdot \left(\frac{\eta }{1‐\eta }\right)>0$$

and

$$N^{***}‐N^{**}=\left(\frac{\theta ‐\eta }{1‐\eta }\right)\frac{k_{4}\min\left\{Q,S\right\}p_{0}e^{k_{6}\int \left(\frac{Q‐S}{S}\right)dt}}{k_{2}\left(\lambda +k_{5}\right)}.$$

When the coefficient of cost assumption satisfies the optimal distribution coefficient formula (52), the above equation can be expressed as

$$N^{***}‐N^{**}=\left(\frac{1‐\theta }{2}\right)\frac{k_{4}\min\left\{Q,S\right\}p_{0}e^{k_{6}\int \left(\frac{Q‐S}{S}\right)dt}}{k_{2}\left(\lambda +k_{5}\right)}=N^{***}\cdot \left(\frac{1‐\theta }{2}\right)>0.$$

As 0<η<1, combining Equation (52) shows that $0<\frac{3\theta ‐1}{1+\theta }<1$. According to the calculation, the necessary condition for the above equation is that the coefficient of income distribution θ satisfies $\frac{1}{3}<\theta <1$.

The retailer's effort in the case of centralized decision‐making is shown to be higher than that in the case of the decentralized decision‐making or decision‐making under the incentive strategy. The retailer's effort in the case of decentralized decision‐making is lower than that in the case of decision‐making under the incentive strategy.

**Conclusion 1.** The supplier's effort in the case of centralized decision‐making is higher than that in the case of decentralized decision‐making or decision‐making under the incentive strategy. The supplier's effort in the case of decentralized decision‐making is the same as that in the case of decision‐making under the incentive strategy. When the optimal allocation is implemented, the retailer pays more effort in the case of centralized decision‐making than in the case of decentralized decision‐making or decision‐making under the incentive strategy. In addition, the retailer pays more effort in the case of decision‐making under the incentive strategy than that in the case of decentralized decision‐making.

### 4.2 Food quality comparison in different situations

According to Equations (33), (58), and (74), food quality in different situations can be compared as follows:

$$R^{**}\left(t\right)‐R^{*}\left(t\right)=\left(\frac{\eta }{1‐\eta }\right)e^{‐k_{5}t}\frac{\left(1‐\theta \right)k_{4}^{2}}{k_{2}\left(\lambda +k_{5}\right)}\int \min\left\{Q,S\right\}p_{0}e^{k_{6}\int \left(\frac{Q‐S}{S}\right)dt+k_{5}t}dt>0$$

and

$R^{***}\left(t\right)‐R^{**}\left(t\right)=\left[\frac{{\left(\theta ‐1\right)}^{2}}{\left(1‐\eta )(1+\theta \right)}\right]e^{‐k_{5}t}\left(\frac{k_{4}^{2}}{\lambda k_{2}+k_{2}k_{5}}\right)\int \min\left\{Q,S\right\}p_{0}e^{k_{6}\int \left(\frac{Q‐S}{S}\right)dt+k_{5}t}dt>0.$.

Then,$R^{***}\left(t\right)>R^{**}\left(t\right)>R^{*}\left(t\right)$ can be obtained through the above comparison.

**Conclusion 2.** Regardless of the market supply and demand relationship, food quality is the highest in the case of centralized decision‐making. The quality of food in the case of decision‐making under the incentive strategy is higher than that in the case of decentralized decision‐making. The quality of food is the worst in the case of decentralized decision‐making. It can be believed that from the situation of decentralized decision‐making to the situation of decision‐making under the incentive strategy and then the situation of centralized decision‐making, a improvement occurs in the food quality.

### 4.3 Comparison of the retailer's and supplier's optimal revenue and the supply chain's overall revenue in different situations

According to Equations (28) and (53), the supplier's optimal revenue in the case of decentralized decision making and decision‐making under the incentive strategy can be compared as follows:

$$V_{M}^{**}\left(R\right)‐V_{M}^{*}\left(R\right)=\frac{\theta \min\left\{Q,S\right\}p_{0}e^{k_{6}\int \left(\frac{Q‐S}{S}\right)dt}}{\lambda +k_{5}}\left(R^{**}‐R^{*}\right)+\frac{{\left(1‐3\theta \right)}^{2}}{8}\frac{k_{4}^{2}}{\lambda k_{2}}{\left\{\frac{\min\left\{Q,S\right\}p_{0}e^{k_{6}\int \left(\frac{Q‐S}{S}\right)dt}}{\lambda +k_{5}}\right\}}^{2}>0.$$

The optimal revenue of the supplier in the case of decision‐making under the incentive strategy is higher than that in the case of decentralized decision‐making.

According to Equations (29) and (54), the retailer's optimal revenue in the case of decentralized decision‐making and decision‐making under the incentive strategy can be compared as follows:

$$V_{N}^{**}\left(R\right)‐V_{N}^{*}\left(R\right)=\frac{\left(1‐\theta \right)\min\left\{Q,S\right\}p_{0}e^{k_{6}\int \left(\frac{Q‐S}{S}\right)dt}}{\lambda +k_{5}}\left(R^{**}‐R^{*}\right)+\frac{\left(3\theta ‐1)(1‐\theta \right)}{4}\frac{k_{4}^{2}}{k_{2}\lambda }{\left\{\frac{\min\left\{Q,S\right\}p_{0}e^{k_{6}\int \left(\frac{Q‐S}{S}\right)dt}}{\lambda +k_{5}}\right\}}^{2}.$$

As 0<η<1, combining Equation (52) shows that $0<\frac{3\theta ‐1}{1+\theta }<1$. According to the calculation, the necessary condition for the above equation is that the coefficient of income distribution θ satisfies $\frac{1}{3}<\theta <1$ and then $V_{N}^{**}\left(R\right)‐V_{N}^{*}\left(R\right)>0$.

According to Equations (30), (55), and (71), the supply chain's overall revenue in the case of decentralized decision‐making and decision‐making under the incentive strategy can be compared as follows:

$$V^{***}\left(R\right)‐V^{**}\left(R\right)=\frac{\min\left\{Q,S\right\}p_{0}e^{k_{6}\int \left(\frac{Q‐S}{S}\right)dt}}{\lambda +k_{5}}\left(R^{***}‐R^{**}\right)+\left\{\left[{\left(\theta ‐1\right)}^{2}+3\right]\frac{k_{3}^{2}}{8\lambda k_{1}}+\left[{\left(1‐\theta \right)}^{2}+2\theta^{2}\right]\frac{k_{4}^{2}}{8\lambda k_{2}}\right\}{\left\{\frac{\min\left\{Q,S\right\}p_{0}e^{k_{6}\int \left(\frac{Q‐S}{S}\right)dt}}{\lambda +k_{5}}\right\}}^{2}>0$$

and

$$V^{**}\left(R\right)‐V^{*}\left(R\right)=\frac{\min\left\{Q,S\right\}p_{0}e^{k_{6}\int \left(\frac{Q‐S}{S}\right)dt}}{\lambda +k_{5}}\left(R^{**}‐R^{*}\right)+\left[\frac{{\left(1‐3\theta \right)}^{2}}{8}+\frac{\left(3\theta ‐1)(1‐\theta \right)}{4}\right]\frac{k_{4}^{2}}{\lambda k_{2}}{\left\{\frac{\min\left\{Q,S\right\}p_{0}e^{k_{6}\int \left(\frac{Q‐S}{S}\right)dt}}{\lambda +k_{5}}\right\}}^{2}>0.$$

**Conclusion 3.** The retailer's optimal revenue in the case of decision‐making under the incentive strategy is higher than that in the case of decentralized decision‐making and so is that of the supplier. Therefore, from the situation of decentralized decision‐making to the situation of decision‐making under the incentive strategy, it is a Pareto improvement for both the supplier and retailer. Moreover, the revenue of the food supply chain as a whole in the case of decision‐making under the incentive strategy is higher than that in the case of decentralized decision‐making.

## 5 SIMULATION ANALYSIS

To study the evolution of food safety status with time, income distribution coefficient, and market supply and demand changes, the optimal returns and efforts of the supplier and retailer and the overall optimal returns of the supply chain under different decisions were analyzed. The effort, revenue, and food quality evolution of the supplier and retailer under different decision‐making conditions were simulated by computational experiments.

Suppose the value of each parameter in the model is as follows: λ=0.04, $k_{1}=0.5$, $k_{2}=0.5$, $k_{3}=0.6$, $k_{4}=0.4$, $k_{5}=0.02$, and $k_{6}=0.01$, $k_{7}=10$. The initial price of food is $p_{0}=0.5$, and the initial quality of food is $R_{0}=20$.

On the one hand, the rate of change of food demand in China is usually faster than the growth rate of food supply (Sheng & Song, 2019); on the other hand, in a specific food industry that is limited by resources and productivity, the change of food supply is usually slower and smaller than the change of food demand. For the sake of calculation, assume that the amount of food produced is S=0.2, and the amount of food demanded by the market is Q(t), with Q(t) as a function of t going up and down around S=0.2 over time. Furthermore, the market demand Q(t) is a random value with a normal distribution and a mean value of 0.2 and a standard deviation of 0.05.

The coefficient of income distribution θ and the coefficient of cost assumption η are assumed to satisfy the optimal distribution coefficient formula (52), and the coefficient of income distribution θ satisfies $\frac{1}{3}<\theta \leq 1$ when making decisions under the incentive strategy.

### 5.1 Optimal decision analysis of the supplier and retailer in different decision‐making situations

**FIGURE 1:**
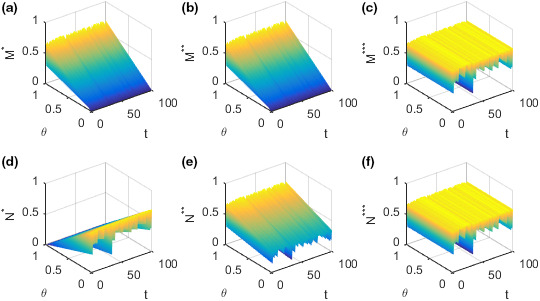
Evolution of the optimal efforts of the supplier and retailer with time and the income distribution coefficients. (a) Plot of the supplier's effort $M^{*}$ in the case of decentralized decision‐making. (b) Plot of the supplier's effort $M^{**}$ in the case of decision‐making under the incentive strategy. (c) Plot of the supplier's effort $M^{***}$ in the case of centralized decision‐making. (d) Plot of the retailer's effort $N^{*}$ in the case of decentralized decision‐making. (e) Plot of the retailer's effort $N^{**}$ in the case of decision‐making under the incentive strategy. (f) Plot of the retailer's effort $N^{***}$ in the case of centralized decision‐making

The evolution of the efforts M and N adopted by the supplier and retailer, respectively, in different situations with the coefficient of income distribution θ and time t is shown in the following Figure 1.

Figure 1(a)–(c) show how the efforts M adopted by the supplier change with time t and income distribution coefficient θ in different decision‐making situations. Figure 1(a) shows how the effort $M^{*}$ adopted by the supplier changes with time t and income distribution coefficient θ in the case of decentralized decision‐making. Figure 1(b) shows how the effort $M^{**}$ adopted by the supplier changes with time t and income distribution coefficient θ in the case of decision‐making under the incentive strategy. Figure 1(c) shows how the effort $M^{***}$ adopted by the supplier changes with time t and income distribution coefficient θ in the case of centralized decision‐making. The efforts $M^{*}$ and $M^{**}$ adopted by the food supplier in the case of decision‐making under decentralized or incentive strategy, respectively, increase with the increase of the income distribution coefficient θ on the whole. However, there is no significant relationship between the effort $M^{***}$ adopted by the food supplier in the case of centralized decision‐making and the income distribution coefficient θ. The effort M adopted by the supplier does not increase and decrease regularly over time t but fluctuates dramatically. Figure 1(d)–(f) show how the efforts N adopted by the retailer changes with time t and income distribution coefficient θ in different decision‐making situations. Figure 1(d) shows how the efforts $N^{*}$ adopted by the retailer changes with time t and income distribution coefficient θ in the case of decentralized decision‐making. Figure 1(e) shows how the efforts $N^{**}$ adopted by the retailer changes with time t and income distribution coefficient θ in the case of decision‐making under the incentive strategy. Figure 1(f) shows how the efforts $N^{***}$ adopted by the retailer changes with time t and income distribution coefficient θ in the case of centralized decision‐making. The effort adopted $N^{*}$ by the food retailer in making decentralized decisions decreases with the increase of income distribution coefficient θ. The effort $N^{**}$ adopted by the food retailer in the case of decision‐making under the incentive strategy increases with the increase of the income distribution coefficient θ. The effort $N^{***}$ adopted by the food retailer in the case of centralized decision‐making is independent of the income distribution coefficient θ. The effort N adopted by the retailer does not increase and decrease regularly over time t but fluctuates dramatically.

**FIGURE 2:**
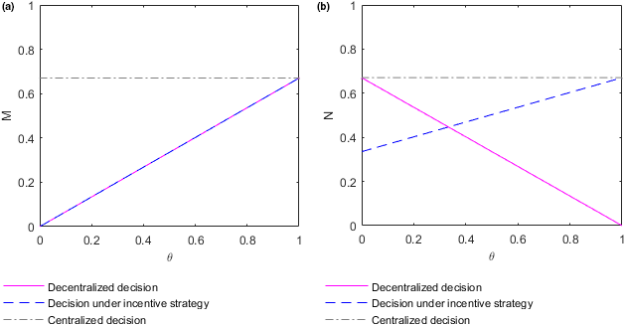
Evolution of the optimal efforts of the supplier and retailer with the income distribution coefficients. (a) Plot of the supplier's effort function M as function in θ. (b) Plot of the retailer's effort function N as function in θ

To better analyze the relationship between the efforts adopted by the supplier and retailer and the income distribution coefficient, take time t=10. At this time, the efforts M and N adopted by the supplier and retailer evolve with the income distribution coefficient θ as shown in the following Figure 2.

Figure 2(a) shows how the effort M adopted by the supplier changes with the income distribution coefficient θ in different decision situations at time t=10. The efforts $M^{*}$ and $M^{**}$ adopted by the food supplier in the case of decision‐making under the decentralized or incentive strategy, respectively, increase with the increase of the income distribution coefficient θ. The reason may be that the supplier can gain more benefits by improving food quality and safety, which increases the enthusiasm of the supplier to improve food quality along with the increase of income distribution coefficient. However, no significant relationship exists between the effort $M^{***}$ adopted by the supplier in the case of centralized decision‐making and the income distribution coefficient θ. The reason may be that both sides of the supply chain make decisions as a whole, and individual income does not affect the overall decision in the case of centralized decision‐making. The effort adopted by the food supplier in the case of centralized decision‐making is higher than that adopted by the food supplier in the case of decentralized decision‐making or incentive strategy. Additionally, the food supplier makes the same efforts in the case of decentralized decision‐making and incentive decision‐making. The results are consistent with **Conclusion 1**. Figure 2(b) shows how the effort N adopted by the retailer changes with the income distribution coefficient θ in different decision situations at time t=10. In the case of decentralized decision‐making, the effort $N^{*}$ adopted by the food retailer decrease with the increase of the income distribution coefficient θ. The reason may be that the retailer can make less profit by trying to improve the quality of food as the distribution coefficient increases, and so the enthusiasm of the retailer to improve food quality decreases with the increase of income distribution coefficient. In the case of decision‐making under the incentive strategy, the effort $N^{**}$ adopted by the food retailer increases with the increase of the income distribution coefficient θ. The reason may be that the food supplier bears part of the cost of food quality efforts for the retailer, so the latter can gain benefits by improving food quality efforts even if the coefficient of income distribution is high. However, with the decrease of the income distribution coefficient, the supplier's efforts in food quality continue to decrease, resulting in the continuous decline of the overall supply chain revenue and, in turn, the failure of the retailer to obtain more revenue through their efforts in improving food quality. As a result, the motivation of the retailer to improve food quality declines with the decrease of income distribution coefficient. In the case of centralized decision‐making, no significant relationship exists between the retailer's effort $N^{***}$ and the income distribution coefficient θ for the same reasons as those for the suppliers. The efforts adopted by the food retailer in the case of centralized decision‐making are higher than those of the food retailer in the case of decision‐making under decentralized or incentive strategies. When the income distribution coefficient satisfies $\theta >\frac{1}{3}$, the effort adopted by the food retailer in the case of decision‐making under the incentive strategy is also higher than that adopted by the food retailer in the case of the decentralized strategy decision‐making. This conclusion is also consistent with **Conclusion 1**.

**FIGURE 3:**
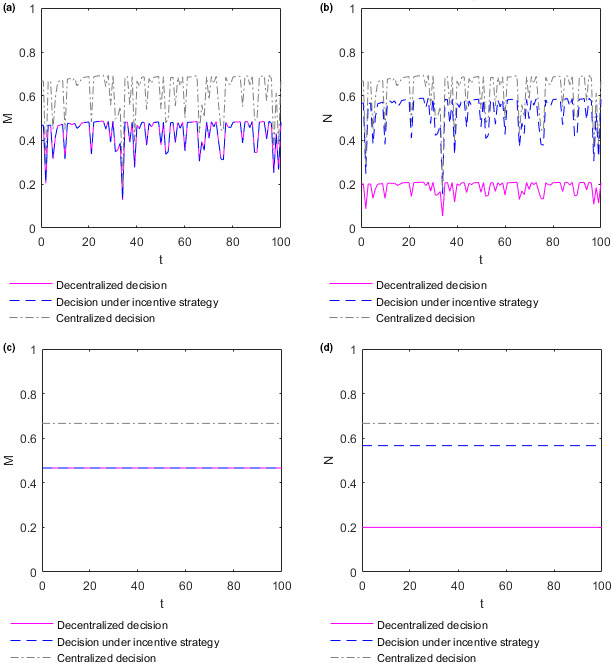
Evolution of the optimal efforts of the supplier and retailer over time. (a) Plot of the supplier's effort function M as function in t when market demand changes randomly over time. (b) Plot of the retailer's effort function N as function in t when market demand changes randomly over time. (c) Plot of the supplier's effort function M as function in t when market demand is constant. (d) Plot of the retailer's effort function N as function in t when market demand is constant

To better analyze the relationship between the efforts adopted by the supplier and retailer and time, take the income distribution coefficient θ=0.7. In this case, the optimal efforts M and N adopted by the supplier and retailer evolve over time t, as shown in Figure 3 below. To distinguish whether the optimal efforts adopted by the food supplier and retailer directly change over time or are influenced by changes in market supply and demand over time, the optimal effort chart of the supplier and retailer over time t with the market demand constant as 0.2 is created, as shown in Figure 3 below, for comparative analysis. When the market demand is constant at 0.2, the optimal efforts of the supplier and retailer are denoted as $M^{C}$ and $N^{C}$, respectively.

Figure 3(a) shows how the optimal effort M adopted by the supplier varies over time t in different decision situations when market demand changes randomly over time. Figure 3(b) shows how the optimal effort N adopted by the retailer varies over time t in different decision situations when market demand changes randomly over time. The optimal efforts M and N adopted by the supplier and retailer fluctuate dramatically over time t, and in most cases fluctuates toward low effort. In the same market supply and demand environment, the relationship between the optimal efforts adopted by the supplier and retailer in different decision situations is consistent with **Conclusion 1**. Figure 3(c) shows how the optimal effort $M^{C}$ adopted by the supplier varies over time t in different decision situations when market demand is constant. Figure 3(b) shows how the optimal effort $N^{C}$ adopted by the retailer varies over time t in different decision situations when market demand is constant. When the market demand is constant at 0.2, the optimal efforts $M^{C}$ and $N^{C}$ adopted by the supplier and retailer do not change over time t. Therefore, the optimal efforts adopted by the food supplier and retailer do not change directly over time but are subject to fluctuations in market supply and demand over time.

The change in market supply and demand environment has a great influence on the optimal decision of the food supplier and retailer, and this influence is more likely to make their optimal decision more inclined to the direction of low effort. The fluctuation of effort adopted by the retailer is the most drastic when decision‐making is centralized, followed by when decision‐making is under the incentive strategy, and lowest when decision‐making is decentralized. Moreover, the fluctuation of effort adopted by the supplier is the most drastic when decision‐making is centralized and lowest when decision‐making is under the decentralized or incentive strategy. However, this finding does not prove that decentralized decision‐making can mitigate these fluctuations because the optimal efforts of the retailer and supplier are the lowest in the case of decentralized decision‐making.

### 5.2 Evolution track analysis of food quality in different decision‐making situations

**FIGURE 4:**
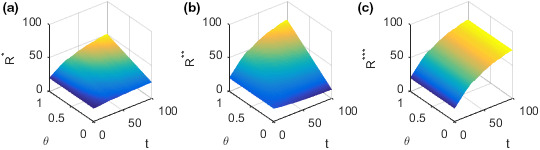
Evolution of food quality with time and the income distribution coefficients. (a) Plot of the food quality $R^{*}$ in the case of decentralized decision‐making. (b) Plot of the food quality $R^{**}$ in the case of decision‐making under the incentive strategy. (c) Plot of the food quality $R^{***}$ in the case of centralized decision‐making

The evolution of food quality R along with income distribution coefficient θ and time t is shown in the following Figure 4.

Figure 4(a)‐(c) show how the food quality R changes with time t and income distribution coefficient θ in different decision‐making situations. Figure 4(a) shows how the food quality $R^{*}$ changes with time t and income distribution coefficient θ in the case of decentralized decision‐making. Figure 4(b) shows how the food quality $R^{**}$ changes with time t and income distribution coefficient θ in the case of decision‐making under the incentive strategy. Figure 4(c) shows how the food quality $R^{***}$ changes with time t and income distribution coefficient θ in the case of centralized decision‐making. In the case of decentralized decision‐making, food quality $R^{*}$ generally improves with the growth of time t and fluctuates slightly. In the case of decision‐making under the incentive strategy, when the income distribution coefficient θ is low, the food quality $R^{**}$ generally decreases with the growth of time t. When the income distribution coefficient θ is high, the food quality $R^{**}$ on the whole keeps improving with the growth of time t. Moreover, food quality fluctuates slightly over time t. In the case of centralized decision‐making, the food quality $R^{***}$ on the whole improves with the growth of time t and fluctuates slightly. In the case of decision‐making under decentralized or incentive strategies, the higher the income distribution coefficient θ is, the faster the food quality $R^{*}$ or $R^{**}$ will be improved. However, it has no relation with the income distribution coefficient θ and the food quality $R^{***}$ in the case of centralized decision‐making.

**FIGURE 5:**
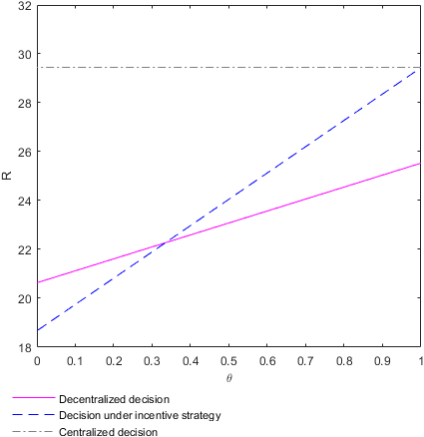
Plot of the food quality function R as function in the income distribution coefficients θ

To better analyze the relationship between food quality and income distribution coefficient, take time t=10. The evolution of food quality R with income distribution coefficient θ is shown in the following Figure 5.

Figure 5 shows how food quality R changes with income distribution coefficient θ in different decision‐making situations at time t=10. In the case of centralized decision‐making, the food quality $R^{***}$ is not affected by the change of income distribution coefficient θ. In the case of decision‐making under the decentralized or incentive strategy, the food quality $R^{*}$ or $R^{**}$ generally increases with the increase of income distribution coefficient θ. The reason may be that the food supplier controls the production and processing links, which are important for food safety assurance, so its efforts have a greater impact on food quality than the efforts of the food retailer. As a result, food quality increases with the increase of income distribution coefficient. In the case of centralized decision‐making, the food quality is the highest. When the income distribution coefficient satisfies $\theta >\frac{1}{3}$, the food quality in the case of decentralized decision‐making is also lower than that in the case of decision‐making under the incentive strategy. The conditions and conclusions here are consistent with **Conclusion 2**.

**FIGURE 6:**
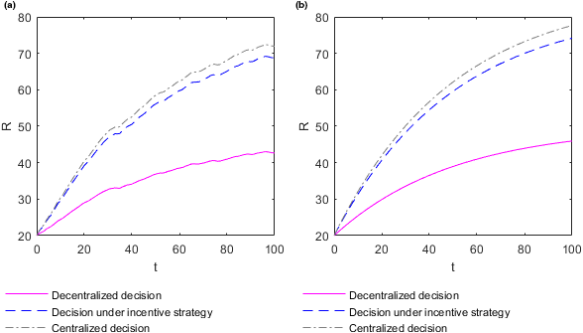
Evolution of food quality over time. (a) Plot of the food quality function R as function in t when market demand changes randomly over time. (b) Plot of the food quality function R as function in t when market demand is constant

To better analyze the relationship between food quality R and time t, take the income distribution coefficient θ=0.7 and the corresponding optimal cost assumption coefficient η=0.647. In this case, the evolution of food quality R over time t is shown in Figure 6(a) below. To distinguish whether changes in food quality over time are affected by changes in market supply and demand, the evolution chart of food quality over time t with the market demand constant as 0.2 is made, as shown in Figure 6(b) below, for comparative analysis. When the market demand is constant at 0.2, the food quality is denoted as $R^{C}$.

Figure 6(a) shows the change of food quality R over time t when market demand changes randomly with time in different decision‐making situations. Food quality R increases with the increase of time t, but the growth rate gradually slows down and fluctuates slightly. In the same market supply and demand environment, the relationship of food quality in different decision‐making situations is consistent with **Conclusion 2**. Figure 6(b) shows the changes of food quality $R^{C}$ over time t in different decision‐making situations when the market demand is constant. When the market demand is constant at 0.2, food quality $R^{C}$ increases with time t, and the growth rate gradually slows down but fluctuates slightly. Therefore, the fluctuation of food quality is influenced by the changes in market supply and market demand over time. In the long term, the random change in market demand leads to slower growth in food quality. In the short term, the random changes in market demand will also lead to a period of decline in food quality.

To sum up, changes in the market supply and demand environment have a certain impact on food quality, most of which are negative for food quality. The difference of food quality fluctuation range between different decision‐making situations is not obvious. Therefore, it can be considered that the change in decision‐making situation cannot reduce the quality fluctuation.

### 5.3 Comparative analysis of the retailer's and supplier's optimal revenue and the supply chain's overall revenue in different decision‐making situations

The optimal revenues $V_{M}$ and $V_{N}$ of the food supplier and retailer, respectively, and the overall revenue of the supply chain V in different decision‐making situations are shown in the following figure along with the evolution of income distribution coefficient θ and time t.

**FIGURE 7:**
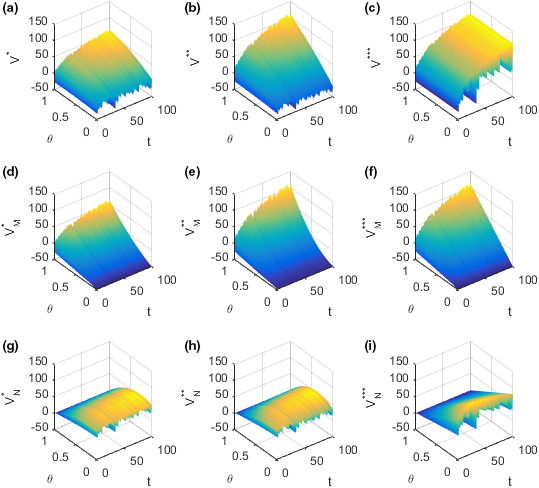
Evolution of optimal revenue of the supplier and retailer and the overall revenue of the supply chain with time and the income distribution coefficients. (a) Plot of the overall revenue $V^{*}$ in the case of decentralized decision‐making. (b) Plot of the overall revenue $V^{**}$ in the case of decision‐making under the incentive strategy. (c) Plot of the overall revenue $V^{***}$ in the case of centralized decision‐making. (d) Plot of the supplier's optimal revenue $V_{M}^{*}$ in the case of decentralized decision‐making. (e) Plot of the supplier's optimal revenue $V_{M}^{**}$ in the case of decision‐making under the incentive strategy. (f) Plot of the supplier's optimal revenue $V_{M}^{***}$ in the case of centralized decision‐making. (g) Plot of the retailer's optimal revenue $V_{N}^{*}$ in the case of decentralized decision‐making. (h) Plot of the retailer's optimal revenue $V_{N}^{**}$ in the case of decision‐making under the incentive strategy. (i) Plot of the retailer's optimal revenue $V_{N}^{***}$ in the case of centralized decision‐making

Figure 7(a)–(c) show how the overall revenue V of the supply chain changes with time t and income distribution coefficient θ in different decision‐making situations. Figure 7(a) shows how the overall revenue $V^{*}$ of the supply chain changes with time t and income distribution coefficient θ in the case of decentralized decision‐making. Figure 7(b) shows how the overall revenue $V^{**}$ of the supply chain changes with time t and income distribution coefficient θ in the case of decision‐making under the incentive strategy. Figure 7(c) shows how the overall revenue $V^{***}$ of the supply chain changes with time t and income distribution coefficient θ in the case of centralized decision‐making. In the case of decentralized decision‐making, the overall revenue $V^{*}$ of the supply chain increases first with the increase of the income distribution coefficient θ and then decreases slowly after reaching the peak value. When the income distribution coefficient θ is high, the overall revenue $V^{*}$ of the supply chain increases with the increase of time t and fluctuates violently. When the income distribution coefficient θ is low with the increase of time t, the trend of overall supply chain revenue $V^{*}$ is not clear and violent fluctuations also occur. In the case of decision‐making under the incentive strategy, the overall revenue $V^{**}$ of the supply chain increases with the increase of income distribution coefficient θ. When the income distribution coefficient θ is high, the overall revenue $V^{**}$ of the supply chain increases with the increase of time t and fluctuates violently. When the income distribution coefficient θ is low, the overall revenue $V^{**}$ of the supply chain decreases with the increase of time t and violent fluctuations also occur. In the case of centralized decision‐making, the overall revenue $V^{***}$ of the supply chain does not have a clear relation with the income distribution coefficient θ, and the trend of overall supply chain revenue $V^{***}$ increases with the increase of time t and fluctuates violently. Figure 7(d)–(f) show how the optimal revenue $V_{M}$ of the supplier changes with time t and income distribution coefficient θ in different decision‐making situations. Figure 7(d) shows how the optimal revenue $V_{M}^{*}$ of the supplier changes with time t and income distribution coefficient θ in the case of decentralized decision‐making. Figure 7(e) shows how the optimal revenue $V_{M}^{**}$ of the supplier changes with time t and income distribution coefficient θ in the case of decision‐making under the incentive strategy. Figure 7(f) shows how the optimal revenue $V_{M}^{***}$ of the supplier changes with time t and income distribution coefficient θ in the case of centralized decision‐making. In the three decision‐making situations, the optimal revenue $V_{M}$ of the supplier increases rapidly with the increase of income distribution coefficient θ. In these three decision situations, the supplier's optimal revenue $V_{M}$ is increased with the increase of time t and violent fluctuations occur. Figure 7(g)–(i) show how the optimal revenue $V_{N}$ of the retailer changes with time t and income distribution coefficient θ in different decision‐making situations. Figure 7(g) shows how the optimal revenue $V_{N}^{*}$ of the retailer changes with time t and income distribution coefficient θ in the case of decentralized decision‐making. Figure 7(h) shows how the optimal revenue $V_{N}^{**}$ of the retailer changes with time t and income distribution coefficient θ in the case of decision‐making under the incentive strategy. Figure 7(i) shows how the optimal revenue $V_{N}^{***}$ of the retailer changes with time t and income distribution coefficient θ in the case of centralized decision‐making. In the case of decision‐making under the decentralized or incentive strategy, the optimal revenue of the retailer $V_{N}^{*}$ first or $V_{N}^{**}$ increases gradually with the increase of income distribution coefficient θ, reaches the peak value, and then decreases gradually. In the case of decision‐making under the centralized strategy, the optimal revenue of the retailer $V_{N}^{***}$ decreases gradually with the increase of income distribution coefficient θ. In the three decision‐making situations, the overall evolution trend of the retailer's optimal revenue $V_{N}$ is not obvious and violent fluctuations also occur.

To better analyze the relationship between food quality and income distribution coefficient, take time t=10. The following figure shows the changes in optimal revenues $V_{M}$ and $V_{N}$ of the supplier and retailer, respectively, and the overall revenue V of the supply chain with income distribution coefficient in different decision‐making situations.

**FIGURE 8:**
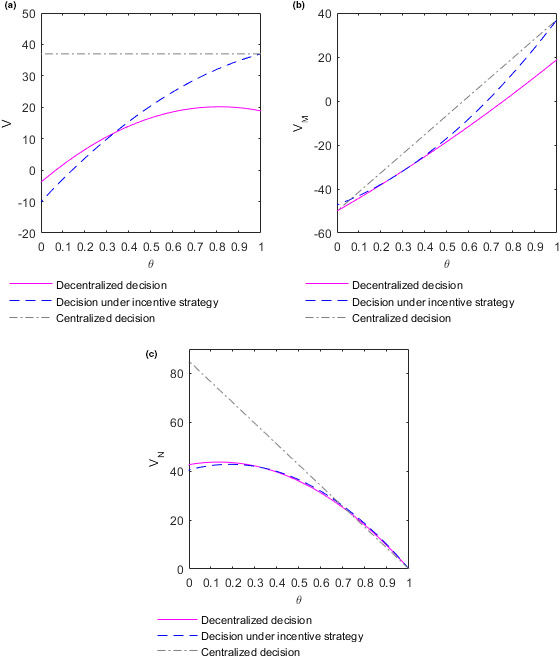
Evolution of optimal revenue of the supplier and retailer and the overall revenue of the supply chain with the income distribution coefficients. (a) Plot of the overall revenue function V as function in θ. (b) Plot of the supplier's optimal revenue function $V_{M}$ as function in θ. (c) Plot of the retailer's optimal revenue function $V_{N}$ as function in θ

Figure 8(a) shows how the overall revenue V of the supply chain changes with the income distribution coefficient θ in different decision situations at time t=10. In the case of centralized decision‐making, the overall revenue $V^{***}$ of the supply chain is not affected by the change of income distribution coefficient θ. In the case of decision‐making under the incentive strategy, the overall revenue $V^{**}$ of the supply chain increases with the increase of income distribution coefficient θ. For the decentralized decision‐making, when θ<0.8, the overall revenue $V^{*}$ of the supply chain keeps increasing with the growth of distribution coefficient θ, but the increase rate gradually slows down and reaches the maximum when θ=0.8. When θ>0.8, the overall revenue $V^{*}$ of the supply chain decreases slowly with the growth of distribution coefficient θ. The reasons may be as follows. In the case of decision‐making under the decentralized or incentive strategy, the quality of the food increases along with the growth of the income distribution coefficient, resulting in increased revenue for the food supply chain. In the case of decision‐making under the incentive strategy, the growth rate of food quality with the income distribution coefficient is large, and the growth rate of income with the income distribution coefficient is always higher than that of cost with income distribution coefficient, so that the overall revenue of food supply chain always increases with the increase of income distribution coefficient. In the case of decentralized decision‐making, the growth rate of food quality with income distribution coefficient is small; when the income distribution coefficient is high, the growth rate of income begins to be lower than the growth rate of cost as the income distribution coefficient increases. Therefore, the overall revenue of the food supply chain decreases slowly with the increase of income distribution coefficient. Given that the quality and cost of food have nothing to do with the income distribution coefficient in the centralized decision‐making process, the total revenue of the supply chain has nothing to do with the income distribution coefficient. In the case of centralized decision‐making, the overall revenue of the supply chain is the highest. When the income distribution coefficient satisfies $\theta >\frac{1}{3}$, the overall revenue of the supply chain in the case of decision‐making under the incentive strategy is also higher than that in the case of decentralized decision‐making, which is consistent with **Conclusion 3**. Figure 8(b) shows how the optimal revenue $V_{M}$ of the supplier changes with the income distribution coefficient θ in different decision situations at time t=10. In the three decision‐making situations, the supplier's optimal revenue $V_{M}$ increases with the increase of distribution coefficient. When the income distribution coefficient satisfies $\theta >\frac{1}{3}$, the optimal revenue of the supplier is the highest in the case of centralized decision‐making. In addition, the optimal revenue of the supplier in the case of decision‐making under the incentive strategy is higher than that in the case of decentralized decision‐making, which is consistent with **Conclusion 3**. Figure 8(c) shows how the optimal revenue $V_{N}$ of the retailer changes with the income distribution coefficient θ in different decision situations at time t=10. In the three decision‐making situations, the retailer's optimal revenue $V_{N}$ decreases with the increase of distribution coefficient θ. When the income distribution coefficient satisfies $\theta >\frac{1}{3}$, the optimal revenue of the retailer in the case of decision‐making under the incentive strategy is higher than that in the case of decentralized decision‐making, which is consistent with **Conclusion 3**.

**FIGURE 9:**
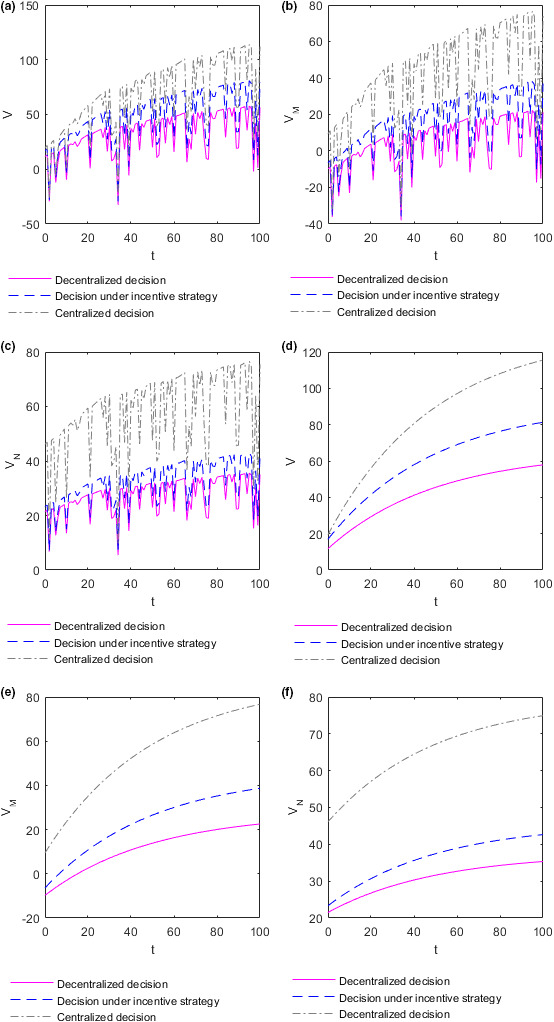
Evolution of optimal revenue of the supplier and retailer and the overall revenue of the supply chain over time. (a) Plot of the overall revenue function V as function in t when market demand changes randomly over time. (b) Plot of the supplier's optimal revenue function $V_{M}$ as function in t when market demand changes randomly over time. (c) Plot of the retailer's optimal revenue function $V_{N}$ as function in t when market demand changes randomly over time. (d) Plot of the overall revenue function V as function in t when market demand is constant. (e) Plot of the supplier's optimal revenue function $V_{N}$ as function in t when market demand is constant. (f) Plot of the retailer's optimal revenue function $V_{N}$ as function in t when market demand is constant

To better analyze the relationship between the optimal revenue of the supplier and retailer and the revenue of the supply chain and income distribution coefficient, take the income distribution coefficient θ=0.7 and the corresponding optimal cost coefficient for η=0.647. In this case, the optimal revenues $V_{M}$ and $V_{N}$ of the supplier and retailer, respectively, and the overall revenue V of the supply chain vary over time, as shown in the Figure 9. To distinguish whether changes in the optimal revenues of the supplier and retailer and the overall revenue of the supply chain over time are affected by changes in market supply and demand, the evolution chart of food quality over time t with the market demand constant as 0.2 is created, as shown in the Figure 9 below, for comparative analysis. When the market demand is constant at 0.2, the optimal revenues of the supplier and retailer are denoted as $V_{M}^{C}$ and $V_{N}^{C}$, respectively, and the overall revenue of the supply chain is denoted as $V^{C}$.

Figure 9(a) shows how the overall revenue V of the supply chain varies over time t in different decision situations when market demand changes randomly over time. Figure 9(b) shows how the optimal revenue $V_{M}$ of the supplier varies over time t in different decision situations when market demand changes randomly over time. Figure 9(c) shows how the optimal revenue $V_{N}$ of the retailer varies over time t in different decision situations when market demand changes randomly over time. The optimal revenues $V_{M}$ and $V_{N}$ of the supplier and retailer, respectively, and the overall revenue of the supply chain increase with the increase of time t, but the growth rate gradually slows down and large fluctuations occur. In the same market supply and demand environment, the relationship between the optimal revenue and the overall revenue of the supply chain of the food supplier and retailer in different decision‐making situations is consistent with **Conclusion 3**. Figure 9(d) shows how the overall revenue V of the supply chain varies over time t in different decision situations when the market demand is constant at 0.2. Figure 9(e) shows how the optimal revenue $V_{M}$ of the supplier varies over time t in different decision situations when the market demand is constant at 0.2. Figure 9(f) shows how the optimal revenue $V_{N}$ of the retailer varies over time t in different decision situations when the market demand is constant at 0.2. When the market demand is constant at 0.2, the optimal revenues $V_{M}^{C}$ and $V_{N}^{C}$ of the supplier and retailer, respectively, and the overall optimal revenue $V^{C}$ of the supply chain increase with time t, and the growth rate gradually slows down without any fluctuations. Therefore, the optimal revenues of the supplier and retailer and the overall revenue of the supply chain are affected by the changes in market supply and market demand over time. The change in the market supply and demand environment has a great impact on the optimal income of the supplier and retailer and the overall revenue of the supply chain. Meanwhile, such impact is more likely to damage the optimal revenues of the food supplier and retailer and the overall revenue of the supply chain. The reason may be that, when the market demand is high, the food enterprises cannot get more profits due to the constant quantity of production, whereas when the market demand is low, the food enterprises will suffer large losses due to their inability to sell the products.

The optimal revenues of the supplier and retailer and overall revenue of the supply chain will fluctuate dramatically due to the change in market supply and demand, and these fluctuations are more likely to result in lower revenues for the supplier and retailer and the overall revenue of the supply chain. These fluctuations are the most drastic when decision‐making is centralized, followed by decision‐making is under the incentive strategy, and the lowest when decision‐making is decentralized. However, this finding does not prove that decentralized decision‐making can mitigate these fluctuations because the retailer and supplier's optimal revenues and the overall revenue of the supply chain are the lowest in the case of decentralized decision‐making.

### 5.4 Analysis of evolution characteristics of food quality

**FIGURE 10:**
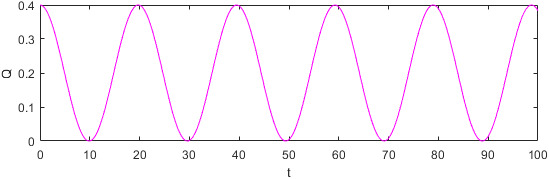
Plot of the food demand function Q as function in time t

To better analyze the evolution characteristics of food quality in different market supply and demand environments, this study analyzes the evolution characteristics of food quality based on the stable market supply and market demand around the fluctuating market supply. It is assumed that under the influence of the market, the change function of food demand over time is $Q=0.2+0.2\cos\frac{t}{\pi }$, as shown in the following figure:

Figure 10 shows that the hypothetical change in food demand over time is consistent with a specific function to explore the evolutionary characteristics of food quality under different food demand changes. In this case, the market demand $Q\left(t\right)$ fluctuates regularly up and down around 0.2 over time t.

**FIGURE 11:**
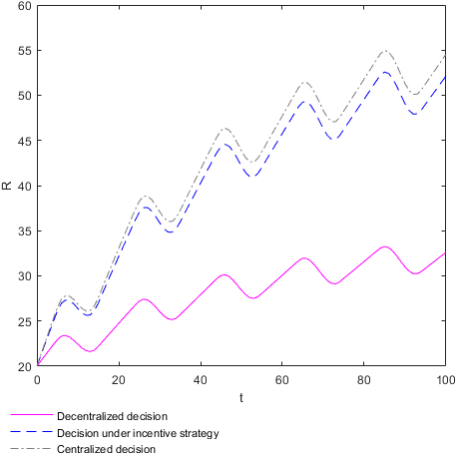
Plot of the food quality function R as function in time t

Figure 11 shows how food quality changes over time as market demand and time fit function $Q=0.2+0.2\cos\frac{t}{\pi }$. The quality of food increases with time, but the quality falls back regularly in a short time due to the fluctuation of market supply and demand.

The supply–demand relationship in Figure 10 illustrates that when the supply exceeds the supply and the market demand is in a downward trend, the food quality gradually improves over time. The maximum value of food quality will likewise be achieved when supply exceeds demand and market demand is on the decline. Then the quality of food will gradually decline over time, and the minimum value of food quality will be reached when supply exceeds demand and market demand is on the rise. When market demand drops from above the market supply to below the market supply and the market demand tends to decline, food quality safety risk is lower than those in other periods, whereas when supply exceeds demand and the market demand turns from a downward trend to an upward trend, food quality safety risk is higher than those in other periods. The reason may be that on the premise of constant market supply, when the market demand is too low and food enterprises are in a state of loss, the food enterprises can only reduce their investment in food quality and safety to reduce costs. Given that food quality is the accumulated result of the efforts of the supplier and retailer, the change in food quality will be a step slower than the change in efforts. Therefore, low‐quality food occurs when supply exceeds demand and market demand is on the rise. Furthermore, at the end of the period when supply exceeds demand and market demand is on the decline, food quality remains high even though companies begin to cut back on spending on food quality.

## 6 CONCLUSION

On the basis of market supply and demand, this study constructs a differential game model between the food supplier and food retailer by considering different decision‐making situations. It analyzes the optimal revenues of the food supplier and food retailer on food quality effort, the overall revenue of the supply chain, and the level of food quality safety and their evolutionary characteristics in different decision‐making situations with market supply and demand change. The research results are as follows:

1. When decision‐making under the incentive strategy, the supplier's and retailer’s optimal revenue, overall revenue of the supply chain, optimal effort of the retailer and supplier, and food quality are the highest all higher than those when decision‐making is decentralized. It can be considered that a Pareto improvement occurs to the food quality strategy of the food supplier, food retailer, and even the whole food supply chain from the situation of decentralized decision‐making to the situation of decision‐making under the incentive strategy. Meanwhile, the food quality and overall revenue of the supply chain are the highest when decision‐making is centralized, followed by when decision‐making is under the incentive strategy, and the lowest when decision‐making is decentralized. It can be considered that an improvement occurs to the food quality and overall revenue of the supply chain from the situation of decentralized decision‐making to the situation of decision‐making under the incentive strategy and to the situation of centralized decision‐making.
2. The optimal revenues of the supplier and retailer, overall revenue of the supply chain, and optimal effort of the supplier and retailer will fluctuate dramatically due to the change in market supply and demand. These fluctuations are more likely to result in less effort on the part of the supplier and retailer and lower revenues for the supplier and retailer and the overall revenue of the supply chain. The evolution of food quality in different decision‐making situations will fluctuate slightly due to the change in market supply and demand, and these fluctuations are more likely to degrade food quality. The change in decision‐making situations cannot reduce these fluctuations.
3. Food quality shows an overall trend of improvement over time, and all of them have a small fluctuation due to the changes in market supply and demand. The fluctuation range is uncertain. In the market supply and demand environment with stable supply and fluctuating demand, when market demand drops from above the market supply to below the market supply and the market demand tends to decline, food quality safety risk is lower than those in other periods, whereas when supply exceeds demand and the market demand turns from a downward trend to an upward trend, food quality safety risk is higher than those in other periods.

## CONFLICT OF INTEREST

The authors declare that they have no competing interests.

## Data Availability

The method in this article is computer mathematical simulation. Numerical simulation analysis is the most effective way to test real‐time dynamic data without a large number of empirical validations. The authors simulate to the evolution of food safety status with time, income distribution coefficient, and market supply, and demand changes by using Matlab2016b software. This paper does not have the data that can be obtained because they directly use the plot function of Matlab2016b software to make the images.
